# Characterization of the self-assembly of New Jersey polyomavirus VP1 into virus-like particles and the virus seroprevalence in Japan

**DOI:** 10.1038/s41598-019-49541-y

**Published:** 2019-09-11

**Authors:** Xianfeng Zhou, Huimin Bai, Michiyo Kataoka, Masahiko Ito, Masamichi Muramatsu, Tetsuro Suzuki, Tian-Cheng Li

**Affiliations:** 1 Department of Virology and Parasitology, Hamamatsu University School of Medicine, Shizuoka, 431-3192 Japan; 2 Department of Virology II, National Institute of Infectious Diseases, Musashi-murayama, Tokyo 208-0011 Japan; 3 The Collaboration Unit for Field Epidemiology of the State Key Laboratory for Infectious Disease Prevention and Control, Nanchang Center for Disease Control and Prevention, Nanchang, Jiangxi 330038 P.R. China; 4 Department of Basic Medicine and Forensic Medicine, Baotou Medical College, Baotou, Inner Mongolia 014060 P.R. China; 5 Department of Pathology, National Institute of Infectious Diseases, Musashi-murayama, Tokyo 208-0011 Japan

**Keywords:** Viral epidemiology, Viral vectors

## Abstract

New Jersey polyomavirus (NJPyV) was discovered in 2014 in a pancreatic transplant recipient’s vascular endothelial cells. Here, in the recombinant baculovirus system, VP1 protein of NJPyV expressed in insect cells was processed. The protein self-assembled into virus-like particles (NJPyV-LPs) in a cell-type-dependent manner, and the particles were then released into the culture media. Spherical ~50-nm-dia. NJPyV-LPs of uniform size with morphology resembling that of the native particles of polyomaviruses were purified from the fraction at 1.33 g/cm^3^ in supernatants of VP1-expressing Sf9 cells. We investigated the antigenic properties of purified NJPyV-LPs and performed a VLP-based enzyme immunoassay to determine the age-specific prevalence of NJPyV infection in a general Japanese population aged 1–70 years. The overall seropositivity rate of anti-NJPyV antibodies was only 1.8%. This might be explained by the low circulation of NJPyV in Japan. This is the first report of a large-scale serological survey of NJPyV in Asia (n = 1,050).

## Introduction

The *Polyomaviridae* is a family of small, circular, double-stranded DNA viruses with an average genome size of approx. 5 kbp. The polyomavirus (PyV) genomes are packed into non-enveloped icosahedral capsid particles with diameters of 45–50 nm. The viral capsid is constructed from VP1, VP2, and VP3 proteins. VP1 is the major structural protein that constitutes the external portion of the viral capsid, and VP1 associates with the minor coat proteins VP2 and VP3, which form the interior shell of the capsid. VP1 can assemble into capsid-like structures or virus-like particles (VLPs) consisting of 72 VP1-pentameric capsomeres^1,2^. PyV infection has been confirmed in humans and a wide range of animals^3,4^.

In general, human PyVs can cause persistent infection, and these infections are asymptomatic. However, a PyV infection can cause serious illnesses, especially in immunocompromised individuals. For example, BK virus (BKPyV) infection leads to nephropathy and cystitis in renal transplant recipients^5^. JC virus (JCPyV) is the cause of progressive multifocal leukoencephalopathy (PML) in AIDS patients, and most PML patients nowadays are found among multiple sclerosis patients treated with Natalizumab^5,6^. The Merkel cell polyomavirus (MCPyV) genome is clonally integrated in the majority of the patients with Merkel cell carcinoma, an aggressive neuroendocrine skin tumor that occurs in elderly and immunosuppressed individuals^7,8^.

In the last decade, as a result of improved molecular techniques — in particular unbiased high-throughput DNA sequencing — nine novel human PyVs have been identified^9–16^. Among them, New Jersey polyomavirus (NJPyV) was discovered in 2014 in vascular endothelial cells of a pancreatic transplant recipient^15^. Based on the available sequencing data to date and the results of phylogenetic analyses, NJPyV, classified as an Alpha-polyomavirus, is most closely related to chimpanzee PyVs and bat PyVs^3,15^. The amino acid homologies of the VP1 region of NJPyV against the human PyVs known as BKPyV, JCPyV, and MCPyV are 48%, 48%, and 56%, respectively. To date, little research has been conducted to analyze the virology of NJPyV, including the characterization of the viral proteins that are expressed and processed in cells. Recent studies of the PyV seroprevalence in European populations have demonstrated moderately low positivity (31.4%–57.5%) with anti-NJPyV antibodies in Italy^17^ and very low (~5%) positivity with anti-NJPyV antibodies in the Netherlands^18^. We conducted the present study to (1) investigate the properties of the processing and self-assembly of NJPyV VP1 in a baculovirus expression system and the antigenicity of NJPyV-LPs and (2) determine the age-specific seroprevalence of NJPyV in a Japanese general population by conducting a VLP-based enzyme-linked immunosorbent assay (ELISA).

## Results

### Expression and processing of NJPyV VP1 in insect Sf9 and Tn5 cells

**Figure 1:**
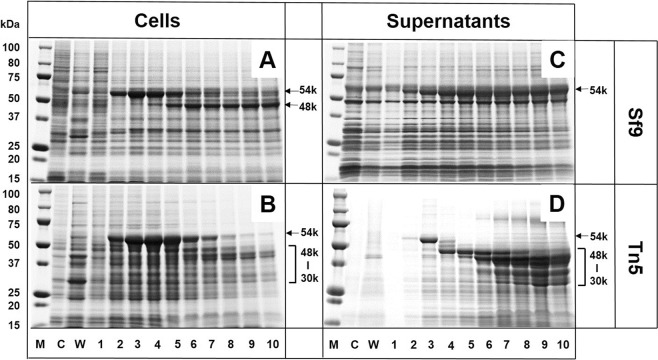
Time course of the expression of NJPyV-VP1 in insect cells. Sf9 and Tn5 cells were respectively infected with Ac[NJPyV-VP1] and harvested at the indicated time periods. SDS-PAGE analyses for (**A**) Sf9 cell lysates, (**B**) Tn5 cell lysates, (**C**) Sf9 culture supernatants, and (**D**) Tn5 culture supernatants are shown. Protein bands were visualized by Coomassie blue staining. Samples from mock-infected- (**C**) and wild-type baculovirus-infected (W) cell cultures were harvested on 3 dpi. M, molecular weight marker. Lanes 1 to 10 = 1 to 10 dpi.

*Spodoptera frugiperda* (Sf9) or BTL-Tn 5B1-4 (Tn5) insect cells were infected with the recombinant baculovirus Ac [NJPyV-VP1] containing NJPyV DNA of the entire VP1 region, followed by the harvesting of the cells and culture supernatants daily until 10 days post-infection (dpi). As indicated by protein gel staining with Coomassie blue, a major protein with a molecular mass of 54 kDa (p54), identical to the predicted size of the entire NJPyV VP1, was detectable from 2 dpi in both cell lines and reached a peak at 3–4 dpi (Supplementary Figs S1 and S2). The amount of the p54 protein declined over time as other proteins were detected: a 48-kDa protein in Sf9 cells and a 30- to 48-kDa protein in Tn5 cells (Fig. 1A,B). In the culture supernatants of infected Sf9 cells, a considerable amount of p54 was detectable at 2–10 dpi (Fig. 1C). In contrast, p54 was detected only at 2–3 dpi in the supernatants of Tn5 culture. A large majority of the proteins observed thereafter appeared to be possible processed forms ranging from 30 to 48 kDa (Fig. 1D).

### Self-assembly and release of NJPyV-LPs in the culture supernatants

**Figure 2:**
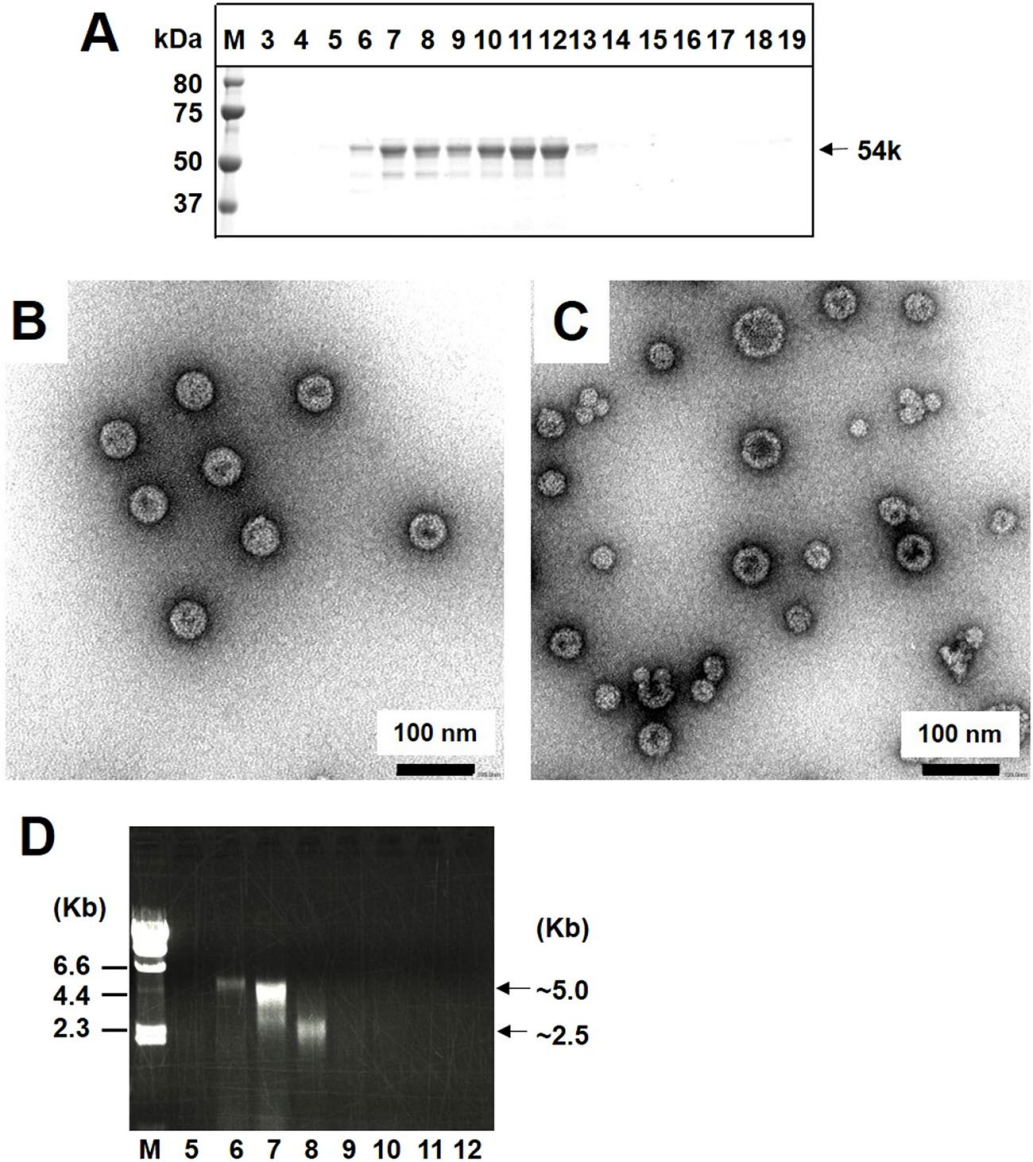
Purification and structural characterization of NJPyV-LP obtained from culture supernatants of NJPyV VP1-expressing Sf9 cells. (**A**) Aliquots from each fraction after CsCl equilibrium density gradient centrifugation of the culture supernatants were analyzed by SDS-PAGE and stained with Coomassie blue. Fraction 7 (**B**) and fraction 12 (**C**) were precipitated, followed by staining with 2% uranyl acetate and observation by TEM. Bars, 100 nm. (**D**) Nucleic acids extracted from fractions 5–12 were analyzed by agarose gel electrophoresis.

The capacity of p54 and its processed forms to assemble into VLPs and their release from the cells was further analyzed. The culture supernatant of Sf9 cells infected with Ac [NJPyV-VP1] was harvested at 7 dpi and subjected to CsCl density gradient centrifugation. The p54 was observed in fractions of 1.29–1.33 g/cm3 (fractions 7–12) (Fig. 2A). Spherical particles of uniform size with ~50 nm dia. and morphology resembling that of native particles of polyomaviruses were detected in fraction 7 (1.33 g/cm3) by transmission electron microscopy (TEM) (Fig. 2B).

In a lower-density fraction, i.e., 1.29 g/cm3 (fraction 12), the expressed NJPyV VP1 was assembled not only into ~50-nm-dia. VLPs but also 20- to 30-nm-dia. VLPs (Fig. 2C). To determine whether nucleic acids derived from host cells or from virus genes were packaged into the NJPyV-LPs, we extracted whole nucleic acids from concentrated CsCl-density fractions and then performed agarose gel electrophoresis. DNA bands (~5 kb and ~2.5 kb, respectively) were detectable in fractions 6–7 and fraction 8 (Fig. 2D, Supplementary Fig. S3). Particle-associated DNAs were not observed in fractions 9–12. It is thus likely that the self-assembly of p54 VP1 together with DNAs into NJPyV-LPs facilitates the stable and efficient formation of ~50-nm-dia. spherical particles in Sf9 cells.

**Figure 3:**
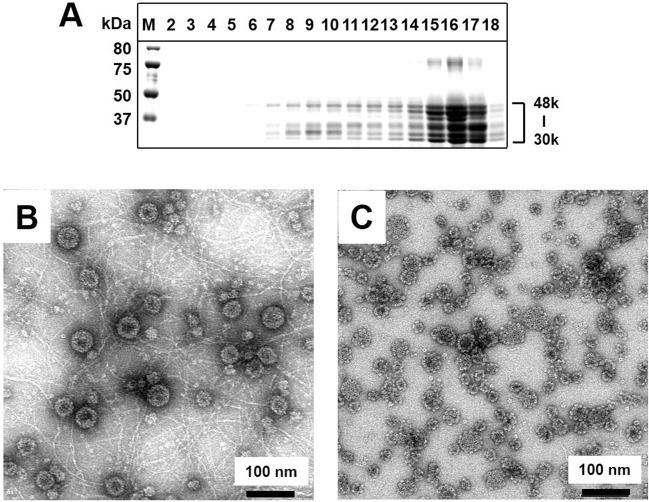
Purification and structural characterization of NJPyV-LP obtained from culture supernatants of NJPyV VP1-expressing Tn5 cells. The culture supernatants were analyzed as described in the Fig. 2 legend. (**A**) Aliquots from each fraction were analyzed by SDS-PAGE and stained with Coomassie blue. Fraction 8 (**B**) and pooled fractions 15–17 (**C**) were precipitated, followed by staining with 2% uranyl acetate and observation by TEM. Bars, 100 nm.

In the same way, we also used the culture supernatant of Ac[NJPyV-VP1]-infected Tn5 cells to determine possible VLP formation by the self-assembly of VP1 in Tn5 cells, in which most of the population was processed to 30–48-kDa proteins (Fig. 3A, Supplementary Fig. S4). The TEM analysis of density fractions prepared as in the case of the Sf9 cultures demonstrated that irregular structures of VLPs ranging from ~20 nm to ~50 nm were observed in fraction 8 with 1.31 g/cm3 (Fig. 3B). A majority of the VLPs in fractions 15–17 with 1.24–1.26 g/cm3 appeared to be assemblies at 20–30-nm in size (Fig. 3C).

Together, the results demonstrated that Sf9 cells but not Tn5 cells are suitable for the efficient expression of p54 VP1 and the subsequent formation of NJPyV-LPs that possess the shape and size comparable to native particles of polyomaviruses, with the use of a baculovirus expression system.

### Limited cross-reactivity of anti-NJPyV serum with antisera against BKPyV, JCPyV, MCPyV and Trichodysplasia spinulosa polyomavirus (TSPyV)

**Figure 4:**
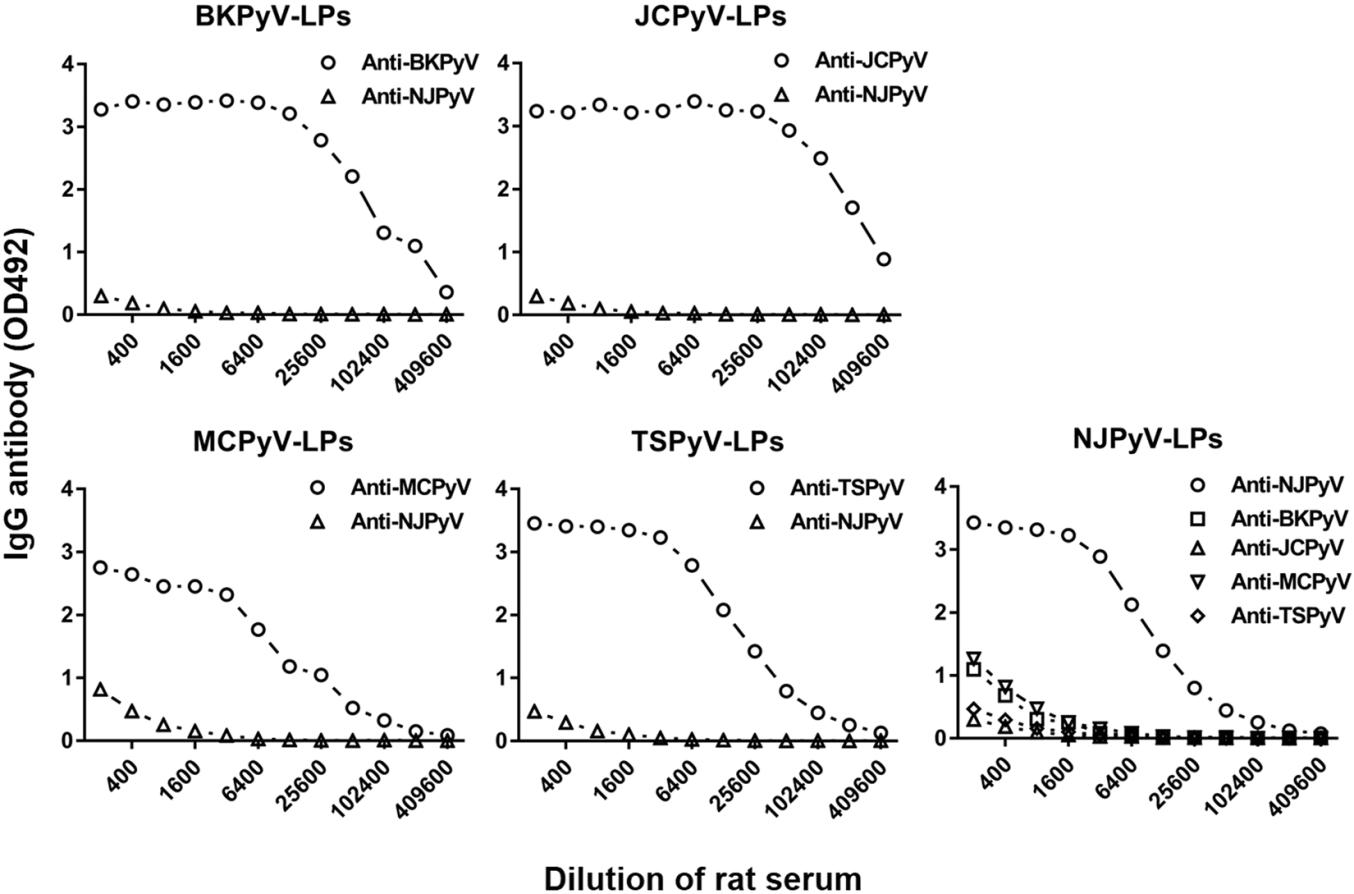
Antigenic cross-reactivity among BKPyV-, JCPyV-, MCPyV-, TSPyV-, and NJPyV-LPs. Hyperimmune sera were obtained from rats that were immunized with BKPyV-, JCPyV-, MCPyV-, TSPyV-, and NJPyV-LPs, respectively. OD values indicating the levels of anti- BKPyV-, JCPyV-, MCPyV- TSPyV-, and NJPyV-LP-IgGs were determined by ELISA.

To test the immune response to NJPyV-LPs prepared from the culture supernatant of Sf9 cells infected with Ac[NJPyV-VP1] (as shown in Fig. 2B) and their antigenic cross-reactivity between human PyVs, we immunized rats with a three-dose intramuscular injection with purified ~50 nm NJPyV-, BKPyV-, JCPyV-, MCPyV- or TSPyV-LPs. All of the immunized rats produced high levels of anti-IgG antibody against each homologous antigen, with titers of 1:409,600 (BKPyV-LPs), 1:819,200 (JCPyV-LPs), 1:204,800 (MCPyV-LPs), 1:409,600 (TSPyV-LPs), and 1:204,800 (NJPyV-LPs), as judged by the results of the antibody ELISA (Fig. 4).

In contrast, the anti-NJPyV-LP antiserum reacted with the heterologous antigens BKPyV-LPs, JCPyV-LPs, MCPyV-LPs, and TSPyV-LPs with the low titers of 1:200, 1:200, 1:800, and 1:400, respectively. We also observed that antisera against BKPyV-LPs, JCPyV-LPs, MCPyV-LPs, or TSPyV-LPs have little potential to be reactive with NJPyV-LPs. It is thus likely that NJPyV does not share its key determinant of antigenicity with BKPyV, JCPyV, MCPyV, or TSPyV.

### Low seroprevalence of anti-NJPyV antibody in a general Japanese population

**Figure 5:**
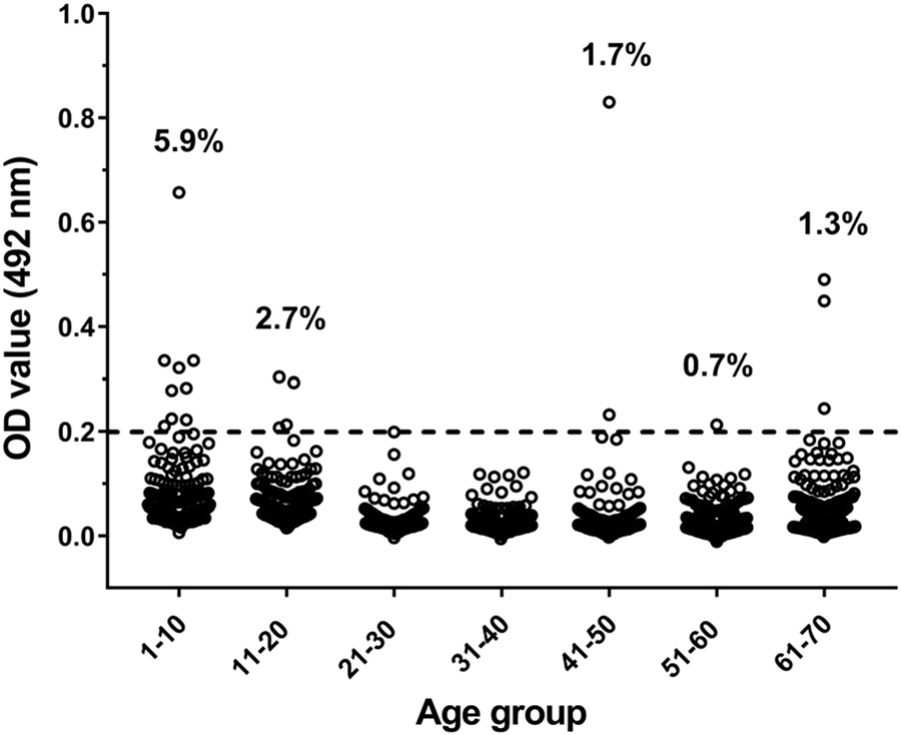
The seropositivity of NJPyV in a Japanese general population (ages 1–70 years). ELISA was conducted coating NJPyV-LPs as the antigen. A *dotted line* represents the cut-off value (OD = 0.2). The NJPyV seropositivity (%) for each age group is indicated. The seropositivity for the 21–30- and 31–40 year-old groups was 0%.

In our previous studies, the VLP-based ELISA was applied to test the age-specific seroprevalence of antibodies against BKPyV, JCPyV, MCPyV, and TSPyV in a general Japanese population aged 1–70 years old^19,20^. The same human serum samples collected from 1,050 individuals (529 males and 521 females) were used for detecting anti-NJPyV IgG antibodies by the ELISA developed in the present study (Fig. 5). To examine the cut-off value for the ELISA, we selected 469 serum samples that were previously indicated as negative for IgG antibodies against at least two human PyVs among BKPyV, JCPyV, and MCPyV. The optical density (OD) values of anti-NJPyV IgG in these serum samples ranged from 0.001 to 0.335. The cut-off value was calculated as 0.20 on the basis of the mean OD plus 3× the standard deviation (SD) (0.05 + 3 × 0.05), Based on the cut-off, only 19 samples were identified as positive for anti-NJPyV antibodies among all samples tested. Thus, in contrast to the cases with BKPyV, JCPyV, MCPyV, and TSPyV (whose seropositivities were 43%–68%^19,20^), the overall prevalence of anti-NJPyV antibodies in the Japanese population was 1.8%. No significant difference in the seroprevalence was observed between the males (2.3%) and females (1.3%).

## Discussion

VLP production via the baculovirus expression system platform in insect cells is a strategy used for the development of vaccines and diagnostic antigens. Although the generation of NJPyV-LPs in insect and yeast cells has been briefly described in recent studies, the questions regarding how the viral protein expressed is processed and assembled had remained unclear. Herein we describe the kinetics of the expression and processing of NJPyV VP1 in two types of insect cells and the characterization of the self-assembled VLPs.

From the comparative analysis of the properties of Sf9 and Tn5 cells, we found that spherical ~50-nm NJPyV-LPs with morphology similar to that of native polyomavirus particles as revealed by TEM can be efficiently and stably produced from Sf9 but not Tn5 cell cultures (Figs 2 and 3). In addition to the entire VP1 p54, several processed forms with molecular masses ranging from 30 to 48 kDa were detectable in both cell lines, whereas most of the VP1 released from Tn5 cells at 4 dpi with Ac[NJPyV-VP1] or later was the processed forms and not p54. Our findings suggest that the proteolytic processing of NJPyV VP1 observed mainly in Tn5 cells may lead to the weakening stability of ~50-nm NJPyV-LP or to lessened efficiency of the assembly of the ~50-nm VLPs. It would be interesting to investigate whether VP1 is similarly processed at the post-translational level in NJPyV-infected cells. It is noted that the reactivity of NJPyV-LPs produced in Tn5 cells with rat anti-NJPyV-LP antiserum was found to be comparable to those produced in Sf9 cells (Supplementary Fig. S5). Although both Sf9 and Tn5 cells are commonly used in combination with baculovirus vectors, it has been reported that the production efficiency and/or post-translational modification(s) of the recombinant proteins are different in cell types depending on the expressed proteins^21,22^.

Our present findings further demonstrated that ~50-nm NJPyV-LPs prepared from culture supernatants of Ac[NJPyV-VP1]-infected Sf9 cells retain immunogenicity that is comparable to the immunogenicity of BKPyV-LPs, JCPyV-LPs, MCPyV-LPs, or TSPyV-LPs but possess low antigenic cross-reactivity against other PyVs (Fig. 4). We therefore applied an ELISA to the NJPyV-LPs to investigate the seroepidemiology of NJPyV in a Japanese population. A variety of studies of the seroprevalence of human PyVs including recently discovered viruses such as MWPyV, STLPyV, and HPyV12 in healthy individuals have been reported^17,18,23^. Although the viral seroprevalence may vary depending on several characteristics such as age, the immunoprofile, and regional features, it is generally accepted that PyVs are ubiquitously distributed; their seroprevalence in adult populations ranges from 40% to 90% in American, European, and Asian countries^18–20,24–26^.

Studies of NJPyV seroprevalence were recently reported in an Italian general population, Dutch blood donors, and immunocompromised transplant recipients^17,18,27^. The seroprevalence of the anti-NJPyV antibodies in an Italian population determined by a VLP-based ELISA showed that the positivities in most of age groups were basically lower compared to other viruses^17^. Kamminga *et al*. reported a very low seroprevalence of NJPyV (~5%) in 18- to 69-year-old subjects as determined by an immunoassay using bacterially expressed VP1 proteins as antigens^18^.

Our present analyses demonstrated that the NJPyV seropositivity in a general population in Japan was markedly low compared to the findings regarding other PyVs in our previous studies^19,20^. The seroprevalence in the 1- to 70-year-old subjects was 1.8%, and that in adulthood (>20 years old) was 0.8% (Fig. 5), indicating a low circulation of NJPyV in Japan. It should be noted that the same population samples were tested for the virus seroprevalence in our previous investigations using a compatible VLP-based ELISA technique; we also observed that the seropositivities of BKPyV, JCPyV, MCPyV, and TSPyV were 68%, 51%, 43%, and 63%, respectively^19,20^. Although there may be variation in the virus prevalence by geographical region, it appears that NJPyV infection is less common compared to most of the human PyVs. Indeed, NJPyV DNA was poorly detectable in sera from close associates of an NJPyV-positive patient, multiple transfusion patients, and nonhuman primates based on the results of polymerase chain reaction analyses^15^.

The present study demonstrated the expression and processing of NJPyV VP1 and its self-assembly into VLPs in insect cells with a recombinant baculovirus and the development of an NJPyV-LP-based ELISA. The NJPyV seroprevalence was only 1.8% in the Japanese general population examined. This is the first report of a large-scale serological screening for the presence of anti-NJPyV antibodies in Asia. Further studies are required to elucidate the geographical diversity of the virus distribution, as well as the transmission mode of NJPyV and its potential involvement in human diseases.

## Materials and Methods

### Construction of recombinant baculovirus and expression of NJPyV VP1

The full-length VP1 DNA of NJPyV containing a *Bam*HI site before the start codon and an *Xba*I site after the stop codon was synthesized based on the viral sequence (GenBank accession no. KF954417). The DNA was then cloned into a baculovirus transfer vector pVL1393 (Pharmingen, San Diego, CA), yielding pVL1393-NJVP1. Sf9 insect cells (Riken Cell Bank, Ibaraki, Japan) were cotransfected with linearized wild-type *Autographa californica* nuclear polyhedrosis virus DNA (BaculoGold 21100D, Pharmingen) and pVL1393-NJVP1 by the lipofectin-mediated method as specified by the manufacturer (Gibco-BRL, Grand Island, NY). The recombinant virus was plaque-purified in Sf9 cells and designated as Ac[NJPyV-VP1]. For the expression of NJPyV VP1, Sf9 and Tn5 insect cells were infected with Ac[NJPyV-VP1] at a multiplicity of infection of 10, respectively. The cells were cultured as described^28,29^.

### Purification of NJPyV-LPs

NJPyV-LPs were purified essentially as described^29^. In brief, the culture supernatant of Sf9 or Tn5 cells infected with Ac[NJPyV-VP1] was spun at 32,000 rpm for 3 hr in a Beckman SW32Ti rotor. The pellet was resuspended in 4.5 ml of EX-CELL^®^ 405 serum-free medium and mixed with 2.1 g of CsCl, followed by centrifugation at 35,000 rpm for 24 hr at 10 °C in a Beckman SW55Ti rotor. Fractions at appropriate densities after the gradient centrifugation were diluted with EX-CELL 405 and centrifuged for 2 hr at 50,000 rpm in a Beckman TLA55 rotor to precipitate NJPyV-LPs.

### Nucleic acid extraction from NJPyV-LPs

Nucleic acids in the purified NJPyV-LPs were prepared using a MagNA Pure LC Total Nucleic Acid Isolation kit (Roche Diagnostics, Indianapolis, IN). Fifty microliters of the extract was treated with DNase I (final concentration; 0.01 mg/ml) (Sigma-Aldrich, St. Louis, MO) at 37 °C for 30 min or with RNase A (final concentration; 0.5 µg/ml) at 37 °C for 1 hr, followed by an analysis on 1% agarose gel electrophoresis.

### TEM

Purified NJPyV-LPs were placed on a carbon-coated grid for 45 sec, rinsed with distilled water, stained with a 2% uranyl acetate solution, and examined with a JEM-1400 transmission electron microscope (JEOL, Japan) operating at 80 kV.

### Hyperimmune sera against NJPyV-, BKPyV-, JCPyV-, MCPyV- or TSPyV-LPs

Fifteen-week-old female Wistar rats (Japan SLC, Hamamatsu, Shizuoka) were immunized with NJPyV-, BKPyV-, JCPyV-, MCPyV- or TSPyV-LPs by intramuscular injection with a dose of 100 µg of VLPs per shot. A booster injection and last injection with the same dose were administered at 4 and 6 weeks after the initial injection, respectively. All injections were carried out without any adjuvant. All animal experiments were approved by the Animal Care and Use Committee of the National Institute of Infectious Diseases (NIID) and carried out according to the “Guides for Animal Experiments Performed at NIID”.

### Human serum samples

The research proposal was approved by the National Serum Reference Bank of the National Institute of Infectious Diseases (Tokyo, Japan) and 1,050 human serum samples collected from healthy Japanese individuals were supplied by the bank. We divided the serum samples into seven age categories based on the donors’ ages: 1–10 (n = 152), 11–20 (n = 150), 21–30 (n = 120), 31–40 (n = 120), 41–50 (n = 120), 51–60 (n = 151), and 61–70 (n = 237) years of age.

### Detection of anti-PyV antibody by ELISA

The VLP-based enzyme-linked immunosorbent assay (ELISA) to detect anti-PyV antibody was developed as described^19,20^. Briefly, 96-well microplates were coated with the purified NJPyV-, BKPyV-, JCPyV-, MCPyV- or TSPyV-LPs (0.1 µg/well), followed by washing three times with 10 mM phosphate-buffered saline (PBS) containing 0.05% Tween 20 (PBS-T) and then blocking with 200 µl of 5% skim milk for 1 hr. After diluted serum samples were added (100 µl/well), the plates were incubated at 37 °C for 1 hr and washed three times as described above.

After the addition of horseradish peroxidase (HRP)-conjugated goat anti-human IgG (H + L) or HRP-conjugated goat anti-rat IgG diluted with PBS-T containing 1% skim milk, the plates were incubated at 37 °C for 1 hr and washed four times with PBS-T. The plates were then incubated in the presence of the substrate orthophenylenediamine and H_2_O_2_ for 30 min, followed by the addition of 4 N H_2_SO_4_ to each well. Absorbance was measured at 492 nm.

## Supplementary information

Supplementary information
