# Supplementary material for: Characterization of the self-assembly of New Jersey polyomavirus VP1 into virus-like particles and the virus seroprevalence in Japan
Source: Sci Rep. 2019 Sep 11;9:13085. doi: 10.1038/s41598-019-49541-y (PMC6739320; doi:10.1038/s41598-019-49541-y)
Supplement: Supplementary file 1 — Supplementary information [file 41598_2019_49541_MOESM1_ESM.pdf]

Characterization of the self-assembly of New Jersey polyomavirus VP1 into virus-like particles and the virus seroprevalence in Japan

Xianfeng Zhou[1,2,3], Huimin Bai[4], Michiyo Kataoka[5], Masahiko Ito[1], Masamichi Muramatsu[2], Tetsuro Suzuki[1], Tian-Cheng Li[2]

[1]Department of Virology and Parasitology, Hamamatsu University School of Medicine, Shizuoka 431-3192, Japan

[2]Department of Virology II, National Institute of Infectious Diseases, Musashi-murayama, Tokyo 208-0011, Japan

[3]The Collaboration Unit for Field Epidemiology of the State Key Laboratory for Infectious Disease Prevention and Control, Nanchang Center for Disease Control and Prevention, Nanchang, Jiangxi 330038, P.R. China

[4]Department of Basic Medicine and Forensic Medicine, Baotou Medical College, Baotou, Inner Mongolia 014060, P.R. China

[5]Department of Pathology, National Institute of Infectious Diseases, Musashi-murayama, Tokyo 208-0011, Japan

Supplementary Figure S1

Full-length blots used to generate Fig.1A, C

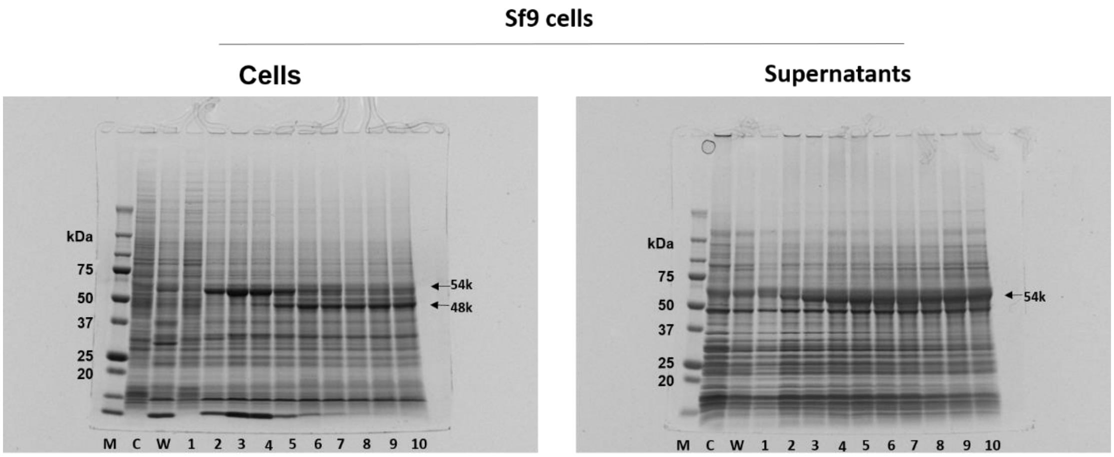

Supplementary Figure S2

Full-length blots used to generate Fig.1B, D

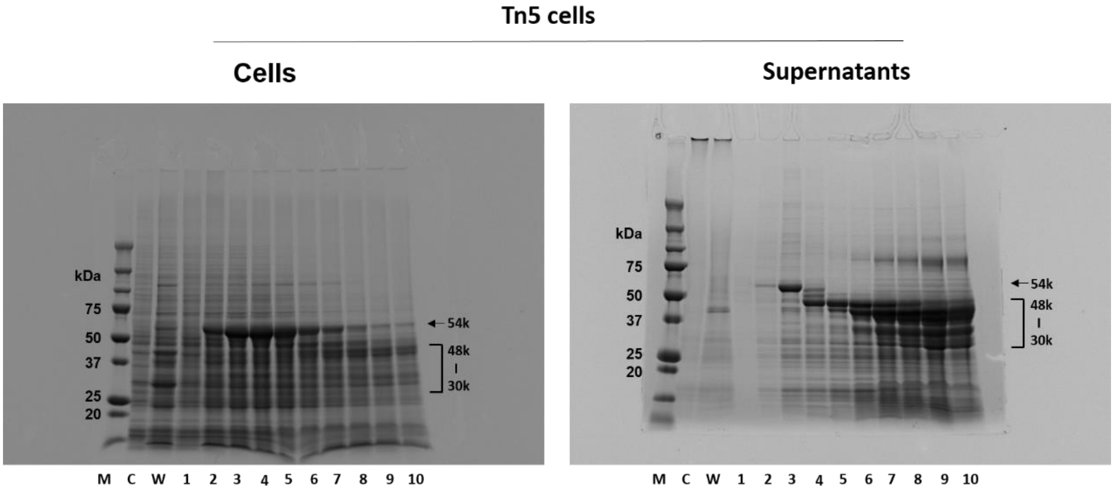

Supplementary Figure S3

Full-length blots used to generate Fig.2A, D

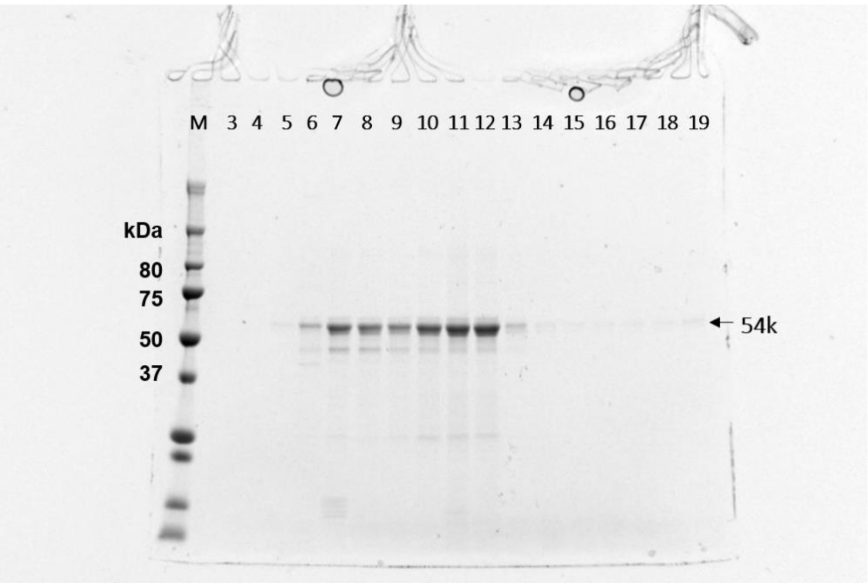

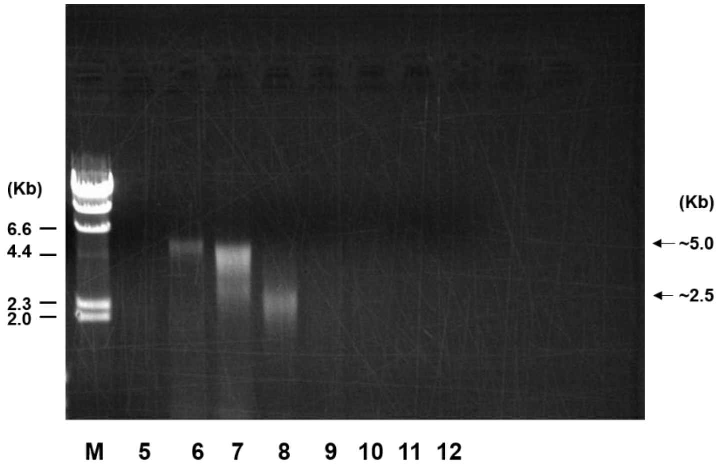

Supplementary Figure S4

Full-length blots used to generate Fig.3A

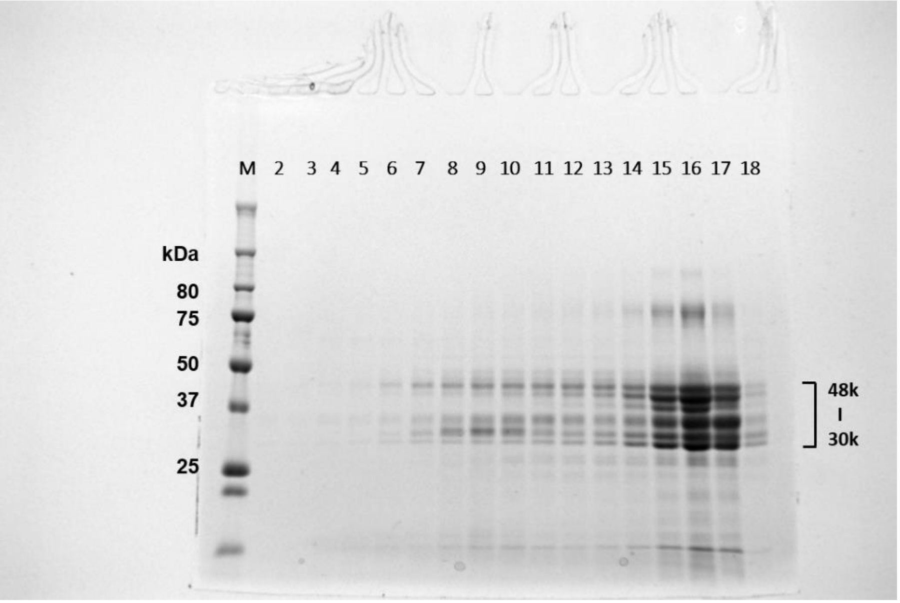

Supplementary Figure S5

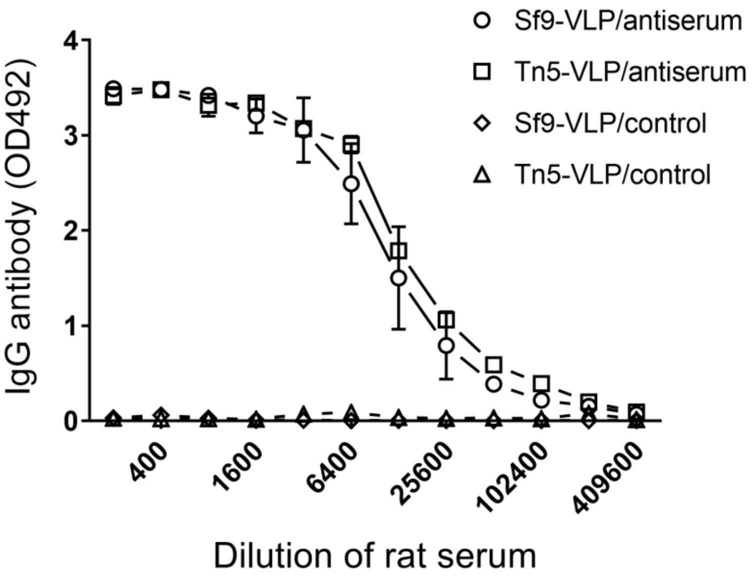

The immune reactivities of NJPyV-LPs produced with Sf9 cells and those produced with Tn5 cells were compared. Microplates were coated with the same masses of NJPyV-LPs, as judged by protein gel staining with Coomassie blue, which were purified from culture supernatants either from Sf9 or Tn5 cells infected with Ac[NJPyV-VP1] (Sf9-VLP or Tn5-VLP). Rat anti-NJPyV serum (antiserum) or serum obtained from pre-immunized rat (control) with a serial dilution was added to each well. The OD values indicating the immune reactivities were plotted (Mean ± SD, n=2).
